# Efficient visual search for facial emotions in patients with major depression

**DOI:** 10.1186/s12888-021-03093-6

**Published:** 2021-02-11

**Authors:** Charlott Maria Bodenschatz, Felix Czepluch, Anette Kersting, Thomas Suslow

**Affiliations:** 1 Department of Psychosomatic Medicine and Psychotherapy, University of Leipzig, Semmelweisstraße 10, 04103 Leipzig, Germany; 2 Department of Social Psychology, University of Leipzig, Neumarkt 9-19, 04109 Leipzig, Germany

**Keywords:** Depression, Visual search, Emotional facial expressions, Attention, Eye-tracking, Gaze behavior, Facial anger, Facial happiness

## Abstract

**Background:**

Major depressive disorder has been associated with specific attentional biases in processing emotional facial expressions: heightened attention for negative and decreased attention for positive faces. However, using visual search paradigms, previous reaction-time-based research failed, in general, to find evidence for increased spatial attention toward negative facial expressions and reduced spatial attention toward positive facial expressions in depressed individuals. Eye-tracking analyses allow for a more detailed examination of visual search processes over time during the perception of multiple stimuli and can provide more specific insights into the attentional processing of multiple emotional stimuli.

**Methods:**

Gaze behavior of 38 clinically depressed individuals and 38 gender matched healthy controls was compared in a face-in-the-crowd task. Pictures of happy, angry, and neutral facial expressions were utilized as target and distractor stimuli. Four distinct measures of eye gaze served as dependent variables: (a) latency to the target face, (b) number of distractor faces fixated prior to fixating the target, (c) mean fixation time per distractor face before fixating the target and (d) mean fixation time on the target.

**Results:**

Depressed and healthy individuals did not differ in their manual response times. Our eye-tracking data revealed no differences between study groups in attention guidance to emotional target faces as well as in the duration of attention allocation to emotional distractor and target faces. However, depressed individuals fixated fewer distractor faces before fixating the target than controls, regardless of valence of expressions.

**Conclusions:**

Depressed individuals seem to process angry and happy expressions in crowds of faces mainly in the same way as healthy individuals. Our data indicate no biased attention guidance to emotional targets and no biased processing of angry and happy distractors and targets in depression during visual search. Under conditions of clear task demand depressed individuals seem to be able to allocate and guide their attention in crowds of angry and happy faces as efficiently as healthy individuals.

**Supplementary Information:**

The online version contains supplementary material available at 10.1186/s12888-021-03093-6.

## Background

Emotional facial expressions (EFEs) represent important and salient features of social interactions. The ability to accurately identify and distinguish between others’ facial expressions is of considerable importance. This holds true especially when multiple stimuli compete for attention, e.g., in a group or a crowd. Successful identification of a face of interest in social settings like these requires guidance of attention as well as the rejection of irrelevant distractors.

While most individuals easily identify and respond to EFEs, individuals with major depressive disorder (MMD) have been found to have difficulties in doing so [1, 2]. For instance, when their task is to indicate the emotional category of a specific facial expression, depressed individuals tend to evaluate positive, neutral, or ambiguous facial expressions as more negative or less positive compared to healthy controls [3].

Depression has further been associated with changes in allocation of attention toward EFEs. A recent review and meta-analysis of eye-tracking studies that analyzed gaze behavior of depressed and healthy individuals found evidence for biased attention [4]. Most of these studies used free-viewing paradigms, in which participants are presented with multiple EFEs at a time and the instruction is to view the pictures freely. Results of these studies indicate increased attention toward negative facial expressions and reduced attention toward positive facial expressions in depressed compared to healthy individuals. Interestingly, between group differences occurred only in attention maintenance to EFEs and not in initial attention orientation [5, 6]. Some authors have speculated that depressed or dysphoric individuals might not orient their attention to negative information more quickly than healthy individuals do, but may exhibit difficulties disengaging from it [7, 8].

The aforementioned free-viewing studies greatly furthered the understanding of depression-related deficits in spontaneous attention allocation toward EFEs. By design, participants in the free-viewing paradigm are not required to allocate their attention in a particular way. Accordingly, it does not allow for an examination of processes of attention to EFEs during active visual search and stimulus comparison.

Visual search paradigms require processes of comparison and search for discrepancies between multiple stimuli. In a variant of the visual search task, multiple stimuli are presented, and participants are instructed to indicate whether all stimuli are from the same category or if one (a “target” stimulus) is different from the others. Visual search efficiency differences have been explained by either differential amounts of guidance provided by a particular target (guidance-based models of visual search [9, 10]), by differences in attention allocation toward distractor stimuli, or by the time it takes to reject distractors [10, 11]. Rinck and Becker [12] used a visual search task to investigate depression-related biases in the processing of emotional words. The authors found no evidence for accelerated detection of depression-related words in the depressed group. However, they found delayed withdrawal of attention from depression-related words in the depressed sample.

A common version of the visual search paradigm is the face-in-the-crowd (FITC) task, which allows the examination of effortful processing of EFEs. To our knowledge, Suslow, Junghanns, and Arolt [13] were the first to use the FITC task to investigate the relationship between depression and spatial processing of facial expressions. The authors exposed their participants to displays of schematic faces with positive and negative emotional expressions and found that depressed individuals had significantly longer reaction times (RTs) in detecting positive faces compared to healthy controls. In their study, the groups did not differ in the detection of negative faces. The authors concluded that depression might be associated with slowed processing of positive facial expressions but not with an abnormal processing of negative facial expressions.

Four further FITC studies have been conducted with depressed and healthy individuals [14–17]. In none of these studies did the groups differ regarding the time needed to respond to positive or negative facial expressions. However, Karparova et al. [14] found generally longer RTs in the depressed compared to the healthy sample. Strand et al. [15] revealed evidence for a specific bias in depressed individuals in response to negative facial expressions. In their study, depressed patients with high symptom load showed longer RTs when the task was to detect a negative face among positive distractor faces. The authors concluded that this effect might indicate a relationship of affective state and a negative processing bias to emotional stimuli.

The inconsistent results of the aforementioned FITC studies might be due to methodological differences in the experimental setup and the sample characteristics. For instance, three of the studies used schematic faces as stimulus material [13, 14, 16]; the other used photographic images of EFEs [15, 17]. In those studies where photographic images were used, this was done to increase ecological validity. It has been argued that photographs of facial expression are preferable to drawings of faces, as they may be more sensitive to depression linked differences [18]. However, while Wisco et al. [17] employed pictures of different individuals in the FITC task, Stand et al. [15] exposed their participants to eight pictures of the same female face in each trial. The repeated use of the same individual in each trial may control for potential perceptual confounds but compromises the ecological validity of a heterogeneous crowd.

The FITC studies mentioned above also varied regarding their sample characteristics. The depressed samples of Karparova et al. [14] and Suslow et al. [16] consisted largely of individuals with comorbid anxiety disorders. With only five exclusively depressed participants in the study of Karparova et al. [14] and only 11 in the study of Suslow et al. [16]. Furthermore, at the time of testing the depressed samples in the studies of Suslow et al. [13] and Strand et al. [15] were to a large extent remitted as indicated by patients’ depression symptom scores.

To summarize, there are only few studies that measured the effects of MDD on processing EFEs while actively searching a visual field, and the results appear to be inconsistent. Furthermore, all aforementioned FITC studies used response latencies as their dependent variable. It has been argued that manual responses do not allow a detailed temporal analysis of attention allocation processes [6]. As mentioned earlier, differences in search latencies may be due to differential amounts of guidance provided by the target, by differences in the time spent on distractors and targets or by differences in the number of distractors fixated. Reaction times do not distinguish between these parameters. A more direct measure of attention is the assessment of eye movements as it provides a spatially and temporally detailed measure of attention allocation. However, it should be noted that recently variants of the traditional reaction-time dot-probe task have been developed that enable a differentiation between processes of attentional engagement and disengagement [19]. The key feature of this approach is that attention is initially anchored in a manner that permits differentially valenced information to be shown either distally or proximally to the attentional focus [20].

Attentional biases for angry facial expression have been shown in depression. Using an emotional variant of the exogenous cueing task it was observed that depressed patients exhibit heightened attention for angry faces suggesting that clinical depression is characterized by increases in attentional processing of negative, interpersonal information [21]. In addition, it was found across reaction-time and eye-tracking indices that remitted depressed patients manifest an increased selective attention toward angry faces, i.e., facial expressions that signal interpersonal rejection [22]. Thus, the attentional bias to angry faces appears to represent a cognitive vulnerability underlying risk for depression recurrence. According to the social risk hypothesis, depression may constitute an adaptive response to the perceived threat of exclusion from social relationships [23]. It was argued that in the ancestral environment depression could have reduced the likelihood of exclusion by heightening perceptual sensitivity to indicators of social threat or disapproval [24].

In the present study, eye movements of clinically depressed patients and healthy controls were tracked during a FITC task. We displayed photographs of facial expressions depicting happiness, anger, and neutral expressions. These emotional categories have been frequently used in previous FITC research finding either superiority effects for happy expressions [25, 26] or superiority effects for angry expressions [27, 28] in healthy individuals. Furthermore, Trapp et al. [29] observed early attentional biases toward happy and angry facial expression in individuals with pronounced depressive mood. To maximize ecological validity, we incorporated photographs of multiple individuals to create more realistic crowds. Comparable to the study of Wisco et al. [17], both female and male faces were included to increase heterogeneity and no individual was presented more than once in each trial. We implemented a mixed design that included every combination of target and distractor.

We hypothesized depression-related biases on attention guidance. That is, in trials with a negative target, depressed individuals should fixate fewer distractor faces prior to the fixation of the target compared to the control group; in trials with a positive target, depressed individuals should fixate more distractor faces prior to the fixation of the target compared to the healthy controls. We further hypothesized an effect of depression-related biases on the time participants spend on the distractor faces. That is, in trials with negative crowds, depressed individuals should fixate distractor faces longer than healthy controls; in trials with positive crowds, depressed individuals should fixate distractor faces more briefly than healthy controls. Finally, we hypothesized an effect of depression-related biases on the time participants spend on the target faces. That is, depressed individuals should fixate negative targets longer and positive targets shorter than healthy controls.

## Methods

### Participants

Thirty-eight (24 female, 14 male) depressed inpatients from the Department of Psychosomatic Medicine and Psychotherapy at the University of Leipzig participated in the study. All patients fulfilled the criteria for a DSM-IV diagnosis of MDD as assessed by the Structured Clinical Interview for the DSM-IV Axis I (SCID; German version [30]) and scored 14 or higher on the revised version of the Beck Depression Inventory (BDI-II; German version, [31]). Patients with other past or present psychiatric conditions and those with distinct symptoms of an anxiety disorder were excluded. The SCIDs were administered by only one interviewer, either Marija Skopinceva (in two thirds of the cases), an experienced clinical psychologist with master’s degree, or by Theresa Ruß, a doctoral student in medicine. Both were trained in the administration of the SCID and later supervised by a senior clinical psychologist (TS). Difficult and ambiguous cases of patients were discussed with the supervisor. Sixty-three percent of the sample were medicated. We examined the effect of medication on eye-tracking parameters and response latencies in our face-in-the-crowd task and report the results as supplementary material. Participants’ mean number of lifetime episodes of MDD was 8.5 (*SD* = 8.2).

Thirty-eight never-depressed healthy control (HC) subjects were selected from a larger sample [32] to match the MDD group for gender. All HC subjects were screened using the Mini-International Neuropsychiatric Interview (M.I.N.I.) [33] to check for exclusion criteria. All M.I.N.I. interviews were conducted by Marija Skopinceva. Subjects with acute symptoms of depression or any history of psychiatric disorder were excluded.

General exclusion criteria for both samples were abuse of alcohol or other substances within the past six months, medical diagnoses associated with neurocognitive impairments as well as the wearing of eyeglasses or contact lenses.

The study was approved by the ethics committee of the University of Leipzig, Medical School, and in accordance with the Declaration of Helsinki. Informed consent was obtained from all subjects prior to the commencement of the study and all participants were financially compensated upon completion of the study.

### Measures

All participants completed a sociodemographic questionnaire (assessing sex, age, and level of education). The BDI-II was administered to assess severity of depressive symptoms. In the present study, the internal consistency (Cronbach’s alpha) of the BDI-II was .74 for the HC sample and .75 for the MDD sample. The State-Trait Anxiety Inventory (STAI; German version [34]) was administrated in its trait form to assess anxious emotional and cognitive reactions. The internal consistency for the STAI was .81 for the HC sample and .90 for the MDD sample. In order to control for possible differences in visual search speed and cognitive flexibility, the Trail Making Test Part B (TMT-B) [35] was administered.

### Stimuli and face-in-the-crowd task

Facial stimuli consisted of 24 photographs of eight actors (four females) selected from the validated Lifespan Database of Adult Emotional Facial Stimuli [36]. Each actor clearly expressed three different emotional qualities: happiness, anger, and neutral. The photographs were processed with Adobe Photoshop to limit each facial expression to the head and neck and to replace background features. All faces were in the same frontal orientation, similar in dimension and gray scaled.

For each trial, eight photographs arranged in a circle were presented simultaneously against a black background. Within the same trial, identities did not repeat, and the positions were randomly assigned. One-third (i.e., 24) of the trials were target absent, i.e., composed of only one emotional category (e.g., all of the eight faces depicted happy expressions). Two-thirds (i.e., 48) were target-present trials, consisting of one face from one (emotional) category and seven faces from a discrepant category (e.g., one happy face among seven angry faces). All target-distractor combinations were utilized (i.e., happy target in angry distractors, happy target in neutral distractors, angry target in happy distractors, angry target in neutral distractors, neutral target in happy distractors, and neutral target in angry distractors). Within the target-present trials, each emotional category appeared once in each of the eight possible positions, resulting in eight trials for each target-distractor combination. The order of all 72 trials was randomized for each participant.

### Procedure

The experiment took place in the eye lab at the Department of Psychosomatic Medicine and Psychotherapy at the University of Leipzig. Participants were tested individually by a trained experimenter. They were seated on an adjustable chair at approximately 70 cm away from the screen. Camera adjustments were made to best capture participants’ eyes and a nine-point grid was used for calibration, followed by a separate validation using the IViewX software. The calibration was repeated if visual deviation was above x/y 0.7°.

Each trial started with the presentation of a fixation cross, shown until a fixation of 1000 ms. Then, the facial stimuli were presented until a response was made or, in case of no response, for 5000 ms.

Participants were instructed on the computer screen that they would see a series of faces composed in a circle and that the task was to press the response button as quickly as possible whenever one of the presented faces differed regarding its emotional expression from the others.

### Eye movement apparatus and parameter

Stimuli were presented on a 22-in. TFT widescreen monitor (resolution: 1680 × 1050) running with an SMI-customized Dell laptop (IView X laptop). Gaze behavior was continuously recorded using an IView X RED250 remote system by SMI, an infrared video-based eye-tracking device sampling eye movements every 4 ms (250 Hz) with a gaze position accuracy of 0.4°. SMIs Experiment Center software was used to present stimuli and to synchronize with recorded eye movements.

Gaze data was computed using a velocity-based algorithm with a minimum fixation duration of 100 ms, a minimum saccade duration of 22 ms, and a peak velocity threshold of 40°/s. BeGaze 3.0 software (SMI, Berlin) was used to define eight areas of interest (AOIs) in each trial corresponding to each of the eight presented facial expressions.

Manual reaction times were assessed, i.e., the time between display onset and button press. Four distinct measures of eye gaze served as dependent variables. First, we wanted to analyze how efficiently participants’ gaze reaches the target as a function of face valence. Therefore, we examined (a) the latency to target face (i.e., time from onset of stimulus display to first fixation on the target). Second, we wanted to examine whether participant groups differed regarding attention guidance to the target face. Therefore, we assessed (b) the number of distractor faces fixated prior to fixating the target. It has been assumed that when a target strongly guides attention, few distractors are selected, and many distractors are skipped. When a target guides attention only weakly, many distractors in the crowd have to be checked before the target is finally found [20]. Third, we wanted to determine whether participant groups differed regarding *distractor processing*. Therefore, we assessed (c) the mean fixation time per distractor face before fixating the target. It has been assumed that, if targets and distractors are similar to each other, or if a stimulus is difficult to perceive, categorization of stimuli as target versus distractor will be more time consuming (see [26]). Finally, we wanted to examine whether patients and controls differed regarding target processing. Therefore, we analyzed (d) the mean fixation time on the target.

The analyses focused on the target present trials. Reaction times and eye-movement measures were analyzed using 6 (condition) × 2 (group) mixed ANOVAs. The Greenhouse Geisser correction [37] was applied to account for violations of sphericity where appropriate.

## Results

As shown in Table 1, study groups did not differ with respect to age, level of education and cognitive flexibility (tested with the TMT-B). However, MDD participants significantly differed from HCs regarding symptoms of depression *t* (47.10) = − 18.70, *p* < .001 and trait anxiety *t* (74) = − 16.41, *p* < .001. Participants in the MDD group reported more symptoms of depression as well as higher levels of anxiety.

**Table 1:** Demographic, affective, and cognitive characteristics of study groups

	MDD (*n* = 38)		HC (*n* = 38)	
	M	SD	M	SD
Age	31.1	7.1	30.1	5.5
Level of education ^a^	2.1	1.0	2.6	1.1
BDI-II (sum score)	24.2	6.7	2.5	2.5
STAI-T (item score)	3.0	0.4	1.6	0.3
TMT-B	64.4	21.1	58.2	16.5

Note: *MDD* depressed participants, *HC* healthy control participants, *M* mean, *SD* standard deviation, ^a^ Coding of level of education:  0 = no degree, 1 = 9th/10th grade, 2 = 12th/13th grade, *3* bachelor degree, *4* master degree, *BDI-II* Beck Depression Inventory-II, *STAI-T* State-Trait Anxiety Inventory, trait version, *TMT-B* Trail Making Test Part B

### Reaction time data

Analyses revealed a significant main effect of condition, *F* (3.92, 290.26) = 168.52, *p* < .001, ${\eta}_p^2$ = .695, but no main effect of group, *F* (1, 74) = .41, *p* = .52, ${\eta}_p^2$ = .006, and no interaction effect, *F* (3.92, 290.26) = .67, *p* = .61, ${\eta}_p^2$ = .009. Independent of study group, participants responded slowest in the conditions neutral target in angry distractors and angry target in neutral distractors (see Table 2).

**Table 2:** Mean reaction times (in ms) as a function of target and crowd emotional expression in the depressed and healthy group

Presentation condition	MDD (*n* = 38)		HC (*n* = 38)	
	M	SD	M	SD
Angry target in happy distractors	1981	306	1944	369
Angry target in neutral distractors	2722	384	2754	459
Happy target in angry distractors	2055	401	2012	406
Happy target in neutral distractors	2121	405	2014	432
Neutral target in angry distractors	2808	430	2742	455
Neutral target in happy distractors	2126	468	2050	350

Note: *MDD* depressed participants, *HC* healthy control participants, *M* mean, *SD* standard deviation

### Eye-movement data

#### Latency to target

Analyses showed a main effect of condition, *F* (4.44, 328.76) = 11.42, *p* < .001, ${\eta}_p^2$ = .134, but no main effect of group, *F* (1, 74) = .05, *p* = .83, ${\eta}_p^2$ = .001, and no interaction effect, *F* (4.44, 328.76) = .71, *p* = .60, ${\eta}_p^2$ = .010. Table 3 shows that, independent of study group, participants’ orientation of gaze to the target face was slowest in the conditions neutral target in angry distractors and angry target in neutral distractors. Permutation-based split-half reliability estimates [38] were obtained separately for each condition, using the split-half package (Version 0.7.1 [39].). The results of 5000 random splits were averaged. Reliability estimates were for latency to target parameters as follows: angry target in happy distractors *r* = 0.47, 95% CI = [0.34, 0.58]; angry target in neutral distractors *r* = 0.57, 95% CI = [0.47, 0.66]; happy target in angry distractors *r* = 0.54, 95% CI = [0.43, 0.63]; happy target in neutral distractors *r* = 0.56, 95% CI = [0.46, 0.65]; neutral target in angry distractors *r* = 0.55, 95% CI = [0.44, 0.64]; and neutral target in happy distractors *r* = 0.64, 95% CI = [0.56, 0.72].

**Table 3:** Mean latencies to target (in ms) as a function of target and crowd emotional expression in the depressed and healthy group

Presentation condition	MDD (*n* = 38)		HC (*n* = 38)	
	M	SD	M	SD
Angry target in happy distractors	1213	181	1208	231
Angry target in neutral distractors	1352	258	1415	336
Happy target in angry distractors	1245	214	1243	287
Happy target in neutral distractors	1313	232	1263	306
Neutral target in angry distractors	1344	254	1367	256
Neutral target in happy distractors	1189	271	1214	240

Note: *MDD* depressed participants, *HC* healthy control participants, *M* mean, *SD* standard deviation

#### Number of distinct distractor faces fixated before fixating the target

A main effect of condition was obtained, *F* (5, 370) = 8.39, *p* < .001, ${\eta}_p^2$ = .102. Independent of study group, participants fixated most distractor faces in the conditions angry target in neutral distractors and neutral target in angry distractors followed by angry target in happy distractors, neutral target in happy distractors, and happy target in neutral distractors. Overall, participants fixated fewest distractor faces in the condition happy target in angry distractors. The ANOVA also revealed a main effect of group, *F* (1, 74) = 4.63, *p* = .03, ${\eta}_p^2$ = .059. While depressed individuals fixated 3.07 (*SD* = 0.58) distinct distractor faces prior to the target face on average, healthy controls fixated *M* = 3.22 (*SD* = 0.62) distractor faces. No interaction effect, *F* (4.44, 328.76) = .71, *p* = .60, ${\eta}_p^2$ = .010, emerged. Table 4 shows the mean number of distractor faces fixated prior to fixating the target by condition and study group. An additional analysis on the effect of medication in the patient group showed that medicated depressed patients fixated fewer distractor faces before fixating the target face compared to unmedicated depressed patients (see supplementary material).

**Table 4:** Mean number of distractor faces fixated prior to fixating the target as a function of target and crowd emotional expression in the depressed and healthy group

Presentation condition	MDD (*n* = 38)		HC (n = 38)	
	M	SD	M	SD
Angry target in happy distractors	3.06	0.53	3.20	0.60
Angry target in neutral distractors	3.19	0.62	3.49	0.72
Happy target in angry distractors	2.82	0.51	3.01	0.64
Happy target in neutral distractors	3.06	0.53	2.98	0.63
Neutral target in angry distractors	3.29	0.66	3.53	0.62
Neutral target in happy distractors	3.00	0.66	3.14	0.63

Note: *MDD* depressed participants, *HC* healthy control participants, *M* mean, *SD* standard deviation

#### Mean fixation time per distractor face before fixating the target

We revealed a main effect of condition, *F* (4.33, 320.16) = 16.22, *p* < .001, ${\eta}_p^2$ = .180, but no main effect of group, *F* (1, 74) = .02, *p* = .90, ${\eta}_p^2$ < .001, and no interaction effect *F* (4.33, 320.19) = .46, *p* = .78, ${\eta}_p^2$ = .006. Independent of study group, participants fixated the distractor faces longest in the condition angry target in neutral distractors and neutral target in angry distractors followed by happy target in neutral distractors, and happy target in angry distractors. Participants fixated the distractor faces shortest in the conditions neutral target in happy distractors and angry target in happy distractors (see Table 5).

**Table 5:** Mean fixation time per distractor face (in ms) before fixating the target as a function of target and crowd emotional expression in the depressed and healthy group

Presentation condition	MDD (*n* = 38)		HC (*n* = 38)	
	M	SD	M	SD
Angry target in happy distractors	651	178	657	191
Angry target in neutral distractors	799	222	848	299
Happy target in angry distractors	708	197	696	241
Happy target in neutral distractors	743	198	724	262
Neutral target in angry distractors	798	212	808	229
Neutral target in happy distractors	661	223	659	207

Note: *MDD* depressed participants, *HC* healthy control participants, *M* mean, *SD* standard deviation

#### Mean fixation time on the target

There was a main effect of condition, *F* (3.70, 273.49) = 105.59, *p* < .001, ${\eta}_p^2$ = .588, but no main effect of group *F* (1, 74) = 1.23, *p* = .27, ${\eta}_p^2$ = .016, and no interaction effect *F* (3.70, 273.49) = .90, *p* = .46, ${\eta}_p^2$ = .012. Independent of study group, participants fixated the target face longest in the condition neutral target in angry distractors followed by angry target in neutral distractors and neutral target in happy distractors (see Table 6). Participants fixated the target faces shortest in the conditions happy target in angry distractors, angry target in happy distractors, and happy target in neutral distractors. Permutation-based split-half reliability estimates were calculated separately for each condition [38, 39]. Reliability estimates were as follows for mean fixation time on target parameters: angry target in happy distractors *r* = 0.52, 95% confidence interval (CI) = [0.40, 0.62]; angry target in neutral distractors *r* = 0.51, 95% CI = [0.40, 0.62]; happy target in angry distractors *r* = 0.51, 95% CI = [0.38, 0.61]; happy target in neutral distractors *r* = 0.54, 95% CI = [0.44, 0.64]; neutral target in angry distractors *r* = 0.58, 95% CI = [0.47, 0.67]; and neutral target in happy distractors *r* = 0.58, 95% CI = [0.48, 0.67].

**Table 6:** Mean fixation time on target (in ms) as a function of target and crowd emotional expression in the depressed and healthy group

Presentation condition	MDD (*n* = 38)		HC (*n* = 38)	
	M	SD	M	SD
Angry target in happy distractors	414	85	383	91
Angry target in neutral distractors	518	115	490	132
Happy target in angry distractors	398	72	399	94
Happy target in neutral distractors	374	80	367	88
Neutral target in angry distractors	641	140	597	144
Neutral target in happy distractors	448	103	436	103

Note: *MDD* depressed participants, *HC* healthy control participants, *M* mean, *SD* standard deviation

### Relationships between eye-tracking parameters and reaction time in the whole sample

For the four eye-tracking parameters considered (latency to target, number of distinct distractor faces fixated before fixating the target, mean fixation time per distractor face before fixating the target, and mean fixation time on the target) and response latency overall means were computed by averaging across face presentation conditions, respectively. Subsequently the correlations between these parameters were calculated. As can be seen in Table 7, on average study participants gave a correct answer after 2.3 s, regardless of face condition. Participants’ gaze entered the target face (i.e., the single discrepant face) 1.3 s after presentation of facial stimuli. Moreover, participants’ gaze entered the target after having fixated on average three distractor faces. The mean fixation duration on the target was less than 0.5 s. Thus, the gaze of participants was on average on the target one second before their button press. Response latency showed high correlations with latency to target, fixation time on distractor before target fixation, and fixation time on target. In general, eye-tracking parameters were strongly correlated. Only number of distractor faces fixated before target fixation and fixation time on target were moderately correlated.

**Table 7:** Descriptive statistics and correlations between overall reaction-time and overall eye-tracking parameters (averaged across face presentation conditions)

	1	2	3	4	5
1. Manual response latency (in ms)	1				
2. Latency to target (in ms)	.72***	1			
3. Number of distinct distractor faces fixated prior to fixating the target	.29*	.67***	1		
4. Mean fixation time per distractor face before fixating the target (in ms)	.65***	.91***	.71***	1	
5. Mean fixation time on the target (in ms)	.81***	.65***	.32**	.68***	1
Mean	2278	1281	3.15	729	456
SD	336	186	0.33	172	79

Note: *N* = 76 for all parameters. * *p* < .05; ** *p* < .01; *** *p* < .001 (two-tailed)

## Discussion

The present study is the first to examine eye movements associated with visual search for emotional and neutral target and crowd faces in depressed and healthy individuals. Eye-tracking methodology has the advantage over reaction-time measures to provide more detailed information on duration and time course of attentional processes during visual perception. In comparison to previous FITC research, we examined not only how fast participants responded to the target faces (by button press), but also how intensely participants’ gaze was guided by the targets as well as allocated on the distractor and target stimuli. Four eye-tracking parameters were analyzed: (a) latency to the target face, (b) number of distinct distractor faces fixated prior to fixating the target, (c) mean fixation time per distractor face before fixating the target and (d) mean fixation time on the target. In our study, we revealed several strong correlations between the eye-tracking parameters. Moreover, response latencies were substantially correlated with latency to target, fixation time on distractor before target fixation, and fixation time on target.

Consistent with previous reaction-time research [15–17], we found no difference between depressed and healthy individuals in their manual response times to the target faces. Both groups responded equally fast in the search task, independent of the emotional category. Contrary to our predictions, we found no alterations in attention guidance and time participants spent on the distractor or target faces. Thus, in the present study depression status neither resulted in a faster attentional guidance in trials with a negative target face nor slower attentional guidance in trials with a positive target face. Depression status further did not result in longer fixation times on the distractor faces in negative crowds nor in shorter fixation times on the distractor faces in positive crowds. Finally, depression status did not result in longer fixation times on negative target faces or in shorter fixation times on positive target faces. Our data indicate that depressed individuals and healthy controls show remarkably similar gaze behavior when processing emotional expressions in crowds of faces under visual search conditions. The normal attentional behavior of depressed patients towards negative social information is noteworthy when considering that previous research suggests heightened attention for angry faces in acute [21] and remitted depression [22]. It has been argued that an attentional bias to angry faces (which indicate social threat or disapproval) might represent a cognitive vulnerability factor implicated in the development and maintenance of depressive disorders [22].

We conducted post-hoc power analyses with the program G*Power 3.1 [40] for the *F*-tests of our main hypotheses, i.e., the interaction of group and condition (ANOVA, repeated measures, within-between interaction) for the parameters *number of distinct distractor faces fixated before fixating the target*, *mean fixation time per distractor face before fixating the target*, and *mean fixation time on the target*. The achieved power in our study to detect a medium-size effect (*f* = .25) given an alpha value of .05, and a total sample size of 76 (with two groups and six measurements) was for the above-mentioned eye-tracking parameters >.95, respectively. The results of additional analyses showed that the power in our investigation to reveal small effect sizes was substantially lower for all eye-tracking parameters (*number of distinct distractor faces fixated before fixating the target*: .38, *mean fixation time per distractor face before fixating the target:* .65, and *mean fixation time on the target:* .61). That is, if the true effect size is small (*f* = .1), only 4 to 6 out of 10 studies should have produced a significant result. Thus, our study was only adequately powered to detect medium or large effects. On the basis of the present findings, it cannot be excluded that depressed patients may differ from healthy individuals to some degree on the gaze parameters considered in our study.

It should further be acknowledged that, independent of study group, a strong effect of valence or valence combination was found in our face-in-the-crowd task. All participants performed worst in the conditions where an angry target was combined with a neutral crowd or a neutral target with an angry crowd. In these conditions, participants needed significantly more time to respond to the target and to find it, they made more and longer fixations on the crowd faces prior to fixating the target, and finally they fixated the target face longer compared to the other conditions. These patterns indicate that it was much more difficult for our participants to find the target when angry and neutral faces were combined. Probably, they had difficulties to differentiate between the two facial expressions. When target and distractor stimuli are similar to each other, the categorization of stimuli as target versus distractor should be more time consuming. It seems unlikely that non-attentional behavioral freezing in the presence of threat faces caused the slowed responding to the stimulus arrays with angry and neutral faces since response times in the conditions angry target in happy distractors and happy target in angry distractors were considerably shorter and similar to those in the conditions neutral target in happy distractors and happy target in neutral distractors. Our results indicating faster processing of happy facial expressions in crowds of faces are in line with findings of other studies suggesting a superiority effect for happy expressions [25, 26]. However, it appears difficult to draw general conclusions on this topic since some studies have reported superiority effects for angry expressions [27, 28] and the pattern of results observed in visual search for emotion faces might largely depend on the specific stimulus materials administered [41].

The task in our study that asked participants to respond when one of the presented faces differed concerning its emotional expression could have encouraged the efficient detection of categorical discrepancies. What kind of processes were applied for the accomplishment of the task? Were elaborative attentional processing and identification of emotional qualities necessary to recognize discrepancies between facial expressions? One part of the answer to these questions could lie in the estimation of task difficulty. In our study, participants’ gaze entered the target, i.e., the single discrepant face, on average 1.3 s after presentation of stimuli. Before target fixation participants have viewed on average at three distractor faces. Correct answers were given on average after 2.3 s, regardless of face condition. That means, that on average one second before button press gaze of participants was on the target. The mean response latency in our study was higher than in many other visual search studies using the face-in-the-crowd task. In investigations administering schematic facial expressions with variable target search as task mean decision latencies were found to be below 1.2 s for faces arranged in 3 × 3 matrices [42] or for crowds of up to 19 faces without fixed positions [43]. Moreover, in studies presenting photographs of faces arranged in 3 × 3 matrices with variable target search as task mean response times were below 1.7 s [28] or below 2.1 s [27] for conditions with happy and angry targets in crowds of emotional or neutral distractors. Thus, in comparison with other studies, level of task difficulty was probably rather high in our study, especially concerning the experimental conditions angry target in neutral distractors and neutral target in angry distractors where our participants responded on average after more than 2.7 s. This suggest that (discrepancy) decisions might have depended, at least in part, upon some kind of elaborative processing. This assumption is also consistent with the observation that, on average, participants fixated three distractor faces before viewing at the target. It appears that some sequential processing of facial stimuli was conducted in our study before a correct decision was reached. In the attentional bias literature in depression, it has been pointed out that depression appears to be characterized by increased elaboration of negative stimuli at later stages of attentional processing [44]. It could be argued that depressive cognitive biases typically emerge when long stimulus presentations enable elaborative processing [45]. However, it should not be overlooked that negative emotional processing biases have also been observed in depressed individuals during the perception of faces that occur automatically, even below the level of conscious awareness [46, 47].

An important question is whether participants identified and compared the emotional quality of the presented facial expression in our face-in-the-crowd task. It is possible that low-level features such as luminance or contrast have driven their visual search [48]. We cannot exclude that our participants based their decisions on visual factors, the perceptual discriminability between targets and distractors, rather than on categorical or affective processing factors. However, there is evidence for facilitated attention capture by angry faces in socially anxious participants during visual search in crowds of faces [49] suggesting that affective disorders can selectively influence visual search performance and direct attention to specific affective qualities. It is remarkable that in our study depressed patients did not allocate more attention to negative, threatening facial expression, regardless of whether it was presented as single target stimulus or as multiple distractor stimulus.

Previous research using free-viewing tasks found evidence for increased attentional maintenance on negative facial expressions and reduced maintenance on positive facial expressions in depressed compared to healthy individuals [4, 50–52]. These results have largely been interpreted as depression-related attentional biases when it comes to EFEs. However, Wisco et al. [17] proposed that results from free-viewing paradigms cannot be interpreted unequivocally as reflecting a depression-related attentional bias, because free-viewing tasks do not place any demands on participants’ attentional pattern. Processing emotional information from facial expressions in everyday life may be influenced by different non-attentional processes. For instance, when one is searching for a specific face or has to distinguish between different faces. Accordingly, people usually scan other faces with a specific goal or task. Wisco et al. [17] assumed that, in the absence of clear task demands (i.e., under free viewing conditions), depressed individuals linger on negative information but are indeed able to withdraw attention from negative information quickly when the task requires doing so. Using a visual search paradigm (in which participants receive a clear instruction), previous behavioral research mostly found no evidence for differences between depressed and healthy individuals in the processing of EFEs [15–17]. However, these results were based on the analyses of RT data, which do not allow a more detailed analysis of attentional processes over time. The present study is the first that used eye movement data to investigate attentional processes in a FITC task. Our results confirm findings from previous RT research, by demonstrating that in a visual search task, depressed individuals process emotional information from multiple facial expressions as efficiently as healthy controls. The discrepant findings between studies using free-viewing paradigms and visual search tasks may underline that the phenomenon of attentional biases in depression is not universal but may emerge only under specific conditions such as viewing freely without specific purpose. In this context it is worth noting that there exists similar evidence of this proposal in the attentional bias literature in anxiety. Anxiety-linked attentional biases to negative stimuli are typically observed in visual search tasks that do not constrain participants’ goals with respect to selective attentional responding. Such tasks permit maximal expression of individual differences in the setting of attentional goals [53].

However, one group difference was revealed. Compared to healthy subjects, depressed individuals fixated fewer distractor faces before fixating the target. This somewhat surprising effect was independent of emotional category. Derakshan et al. [54] proposed that the index *number of crowd faces fixated prior to fixating the target* may indicate vigilant scanning. Accordingly, our depressed participants were less attracted by the distractor faces and scanned the crowds less vigilantly, regardless of valence. The observed group difference is consistent with results from an eye tracking study where depressed and healthy subjects viewed positive and negative word pairs [55]. Participants’ task was to attend to one word (target) and ignore the other one (distractor). In this study, depressed individuals were better able to direct their attention towards target words, regardless of valence, compared to the healthy control group. The authors, who expected depressed individuals to show more difficulty in ignoring the negative distractors (as we did in our study), conclude that the MDD individuals did fairly well in this task. The question remains, why depressed individuals are less attracted by the distractors, while healthy controls display more attention to them. Ellis et al. [55] proposed that the gaze behavior observed in their healthy sample may represent an adaptive attentional process. It was argued that an appropriate processing of continuously changing environmental stimuli, requires attentional flexibility. While depressed individuals compliantly follow task instructions, healthy individuals display modulation of attention. However, in the present study the observed advantage of depressed individuals in the guidance of attention to the target faces did not result in a better performance in other observed parameters. It should be mentioned that our explorative analysis on the effect of medication in the patient group revealed that medicated patients fixated fewer distractors before fixating the target face compared to unmedicated patients. Future research has to further clarify whether antidepressant medication could enhance efficiency of visual scanning in tasks with emotional stimuli, in the sense that distractor stimuli become less often the subject of attention.

Some limitations have to be acknowledged. Only one interviewer conducted the clinical diagnostic interviews (SCID). Therefore, in our study inter-rater reliability could not be examined. Reliability of eye-tracking parameters was quite low for our visual search task so that one must be particularly cautious in the interpretation of our null findings. To obtain higher internal consistencies we suggest increasing the number of trials per condition in future studies administering the face-in-the-crowd task. In the present study, facial stimuli consisted of angry, happy, and neutral facial expressions. Although angry and happy facial expressions have been used regularly in the FITC paradigm [25] the utilization of other facial expressions (e.g., sad facial expression) would be informative. For a comprehensive understanding of effortful processing of emotional information from facial expressions, future research should also include dysphoric faces in the FITC paradigm. Finally, our study is the first that examined gaze behavior of depressed and healthy participants in a FITC task. Although our results fit to previous RT research, it should be interpreted with caution. Further eye-tracking studies are needed before firm conclusions can be reached.

## Conclusions

To summarize, we found no evidence for biased attention guidance to emotional target faces or altered processing of angry and happy distractors and targets in clinical depression during visual search. Under conditions of clear task demand, depressed patients seem to be able to allocate and guide their attention in crowds of angry and happy faces as efficiently as healthy individuals.

## Supplementary Information

**Additional file 1: Table S1**. Reaction time (in ms) as a function of target and crowd emotional expression in unmedicated and medicated depressed participants. **Table S2.** Latency to target (in ms) as a function of target and crowd emotional expression in unmedicated and medicated depressed participants. **Table S3.** Number of distinct distractor faces fixated before target fixation as a function of target and crowd emotional expression in unmedicated and medicated depressed participants. **Table S4.** Mean fixation time (in ms) per distractor face before target fixation as a function of target and crowd emotional expression in unmedicated and medicated depressed participants. **Table S5.** Mean fixation time (in ms) on target as a function of target and crowd emotional expression in unmedicated and medicated depressed participants.

## Data Availability

Data supporting our findings will be shared upon request. If interested, please contact the corresponding author by email (TS).

## Abbreviations

AOI: Area of interest
BDI: Beck Depression Inventory
EFEs: Emotional facial expressions
FITC: Face-in-the-crowd
MDD: Major depressive disorder
M.I.N.I.: Mini-International Neuropsychiatric Interview
RT: Reaction-time
SCID: Structured Clinical Interview for the DSM-IV Axis I
STAI: State-Trait Anxiety Inventory
TMT-B: Trail Making Test Part B

## References

[1] Joormann J, Gotlib IH (2006). Is this happiness I see? Biases in the identification of emotional facial expressions in depression and social phobia. J Abnorm Psychol.

[2] Surguladze SA, Young AW, Senior C, Brébion G, Travis MJ, Phillips ML (2004). Recognition accuracy and response bias to happy and sad facial expressions in patients with major depression. Neuropsychology..

[3] Bourke C, Douglas K, Porter R (2010). Processing of facial emotion expression in major depression: a review. Aust N Z J Psychiatry.

[4] Suslow T, Hußlack A, Kersting A, Bodenschatz CM (2020). Attentional biases to emotional information in clinical depression: a systematic and meta-analytic review of eye tracking findings. J Affect Disord.

[5] Kellough JL, Beevers CG, Ellis AJ, Wells TT (2008). Time course of selective attention in clinically depressed young adults: an eye tracking study. Behav Res Ther.

[6] Sanchez A, Vazquez C, Marker C, LeMoult J, Joormann J (2013). Attentional disengagement predicts stress recovery in depression: an eye-tracking study. J Abnorm Psychol.

[7] Gotlib IH, Joormann J (2010). Cognition and depression: current status and future directions. Annu Rev Clin Psychol.

[8] Koster EHW, de Raedt R, Goeleven E, Franck E, Crombez G (2005). Mood-congruent attentional bias in dysphoria: maintained attention to and impaired disengagement from negative information. Emotion..

[9] Wolfe JM (1994). Guided Search 2.0 A revised model of visual search. Psychonom Bull Rev.

[10] Wolfe JM, Horowitz TS (2017). Five factors that guide attention in visual search. Nat Hum Behav.

[11] Horstmann G, Becker S, Ernst D (2017). Dwelling, rescanning, and skipping of distractors explain search efficiency in difficult search better than guidance by the target. Vis Cogn.

[12] Rinck M, Becker ES (2005). A comparison of attentional biases and memory biases in women with social phobia and major depression. J Abnorm Psychol.

[13] Suslow T, Junghanns K, Arolt V (2001). Detection of facial expressions of emotions in depression. Percept Mot Skills.

[14] Karparova SP, Kersting A, Suslow T (2005). Disengagement of attention from facial emotion in unipolar depression. Psychiatry Clin Neurosci.

[15] Strand M, Sætrevik B, Lund A, Hammar Å (2013). The relationship between residual symptoms of depression and emotional information processing. Nord J Psychiatry.

[16] Suslow T, Dannlowski U, Lalee-Mentzel J, Donges U-S, Arolt V, Kersting A (2004). Spatial processing of facial emotion in patients with unipolar depression: a longitudinal study. J Affect Disord.

[17] Wisco BE, Treat TA, Hollingworth A (2012). Visual attention to emotion in depression: facilitation and withdrawal processes. Cogn Emot.

[18] Gotlib IH, Kasch KL, Traill S, Joormann J, Arnow BA, Johnson SL (2004). Coherence and specificity of information-processing biases in depression and social phobia. J Abnorm Psychol.

[19] Rudaizky D, Basanovic J, MacLeod C (2014). Biased attentional engagement with, and disengagement from, negative information: Independent cognitive pathways to anxiety vulnerability. Cogn Emot.

[20] Clarke PJF, MacLeod C, Guastella AJ (2013). Assessing the role of spatial engagement and disengagement of attention in anxiety-linked attentional bias: a critique of current paradigms and suggestions for future research directions. Anxiety Stress Coping.

[21] Leyman L, De Raedt R, Schacht R, Koster EHW (2007). Attentional biases for angry faces in unipolar depression. Psychol Med.

[22] Woody ML, Owens M, Burkhouse KL, Gibb BE (2016). Selective attention toward angry faces and risk for major depressive disorder in women: converging evidence from retrospective and prospective analyses. Clin Psychol Sci.

[23] Allen NB, Badcock PBT (2003). The social risk hypothesis of depressed mood: evolutionary, psychosocial, and neurobiological perspectives. Psychol Bull.

[24] Allen NB, Badcock PBT (2006). Darwinian models of depression: a review of evolutionary accounts of mood and mood disorders. Prog Neuro-Psychopharmacol Biol Psychiatry.

[25] Becker DV, Anderson US, Mortensen CR, Neufeld SL, Neel R (2011). The face in the crowd effect unconfounded: happy faces, not angry faces, are more efficiently detected in single- and multiple-target visual search tasks. J Exp Psychol Gen.

[26] Horstmann G, Becker SI (2020). More efficient visual search for happy faces may not indicate guidance, but rather faster distractor rejection: evidence from eye movements and fixations. Emotion..

[27] Pinkham AE, Griffin M, Baron R, Sasson NJ, Gur RC (2010). The face in the crowd effect: anger superiority when using real faces and multiple identities. Emotion..

[28] Shasteen JR, Sasson NJ, Pinkham AE (2014). Eye tracking the face in the crowd task: why are angry faces found more quickly. PLoS One.

[29] Trapp W, Kalzendorf C, Baum C, Hajak G, Lautenbacher S (2018). Attentional biases in patients suffering from unipolar depression: results of a dot probe task investigation. Psychiatry Res.

[30] Wittchen H-U, Wunderlich U, Gruschwitz S, Zaudig M (1997). Strukturiertes Klinisches Interview für DSM-IV. Achse I: Psychische Störungen. Interviewheft und Beurteilungsheft. Eine deutschsprachige, erweiterte Bearbeitung der amerikanischen Originalversion des SKID-I.

[31] Hautzinger M, Keller F, Kühner C (2006). BDI-II. Beck depressions-Inventar revision. Frankfurt am main.

[32] Bodenschatz CM, Skopinceva M, Kersting A, Quirin M, Suslow T (2018). Implicit negative affect predicts attention to sad faces beyond self-reported depressive symptoms in healthy individuals: an eye-tracking study. Psychiatry Res.

[33] Ackenheil M, Stotz-Ingenlath G, Dietz-Bauer R, Vossen A (1999). MINI Mini international neuropsychiatric interview. German version 5.0. 0 DSM IV.

[34] Laux L, Glanzmann P, Schaffner P, Spielberger CD (1981). Das State-Trait-Angstinventar. Theoretische Grundlagen und Handanweisungen.

[35] Reitan RM (1992). Trail Making Test.

[36] Ebner NC, Riediger M, Lindenberger U (2010). FACES--a database of facial expressions in young, middle-aged, and older women and men: development and validation. Behav Res Methods.

[37] Greenhouse SW, Geisser S (1959). On methods in the analysis of profile data. Psychometrika..

[38] Parsons S, Kruijt AW, Fox E (2019). Psychological science needs a standard practice of reporting the reliability of cognitive-behavioral measurements. Adv Methods Pract Psychol Sci.

[39] Parsons S. Split half: robust estimates of split half reliability. figshare. Software. 2020. 10.6084/m9.figshare.11956746.v4.

[40] Faul F, Erdfelder E, Buchner A, Lang AG (2009). Statistical power analyses using G*power 3.1: tests for correlation and regression analyses. Behav Res Methods.

[41] Savage RA, Lipp OV, Craig BM, Horstmann G (2013). In search of the emotional face: Anger versus happiness superiority in visual search. Emotion.

[42] Öhman A, Lundqvist D, Esteves F (2001). The face in the crowd revisited: A threat advantage with schematic stimuli. J Pers Soc Psychol.

[43] Eastwood JD, Smilek D, Merikle PM (2001). Differential attentional guidance by unattended faces expressing positive and negative emotion. Percept Psychophys.

[44] Foland-Ross LC, Gotlib IH (2012). Cognitive and neural aspects of information processing in major depressive disorder: an integrative perspective. Front Psychol.

[45] Bistricky SL, Ingram RE, Atchley RA (2011). Facial affect processing and depression susceptibility: cognitive biases and cognitive neuroscience. Psychol Bull.

[46] Victor TA, Furey ML, Fromm SJ, Öhman A, Drevets WC (2010). Relationship between amygdala responses to masked faces and mood state and treatment in major depressive disorder. Arch Gen Psychiatry.

[47] Stuhrmann A, Dohm K, Kugel H, Zwanzger P, Redlich R, Grotegerd D (2013). Mood-congruent amygdala responses to subliminally presented facial expressions in major depression: associations with anhedonia. J Psychiatry Neurosci.

[48] Nummenmaa L, Calvo MG (2015). Dissociation between recognition and detection advantage for facial expressions: a meta-analysis. Emotion..

[49] Eastwood JD, Smilek D, Oakman JM, Farvolden P, van Ameringen M, Mancini C (2005). Individuals with social phobia are biased to become aware of negative faces. Vis Cogn.

[50] Duque A, Vázquez C (2015). Double attention bias for positive and negative emotional faces in clinical depression: evidence from an eye-tracking study. J Behav Ther Exp Psychiatry.

[51] Soltani S, Newman K, Quigley L, Fernandez A, Dobson K, Sears C (2015). Temporal changes in attention to sad and happy faces distinguish currently and remitted depressed individuals from never depressed individuals. Psychiatry Res.

[52] Armstrong T, Olatunji BO (2012). Eye tracking of attention in the affective disorders: a meta-analytic review and synthesis. Clin Psychol Rev.

[53] Basanovic J, MacLeod C (2017). Does anxiety-linked attentional bias to threatening information reflect bias in the setting of attentional goals, or bias in the execution of attentional goals?. Cogn Emot..

[54] Derakshan N, Koster EHW (2010). Processing efficiency in anxiety: evidence from eye-movements during visual search. Behav Res Ther.

[55] Ellis AJ, Wells TT, Vanderlind WM, Beevers CG (2014). The role of controlled attention on recall in major depression. Cogn Emot..

