# Supplementary material for: Efficient visual search for facial emotions in patients with major depression
Source: BMC Psychiatry. 2021 Feb 11;21:92. doi: 10.1186/s12888-021-03093-6 (PMC7879523; doi:10.1186/s12888-021-03093-6)
Supplement: Supplementary file 1 — Additional file 1: Table S1. Reaction time (in ms) as a function of target and crowd emotional expression in unmedicated and medicated depressed participants. Table S2. Latency to target (in ms) as a function of target and crowd emotional expression in unmedicated and medicated depressed participants. Table S3. Number of distinct distractor faces fixated before target fixation as a function of target and crowd emotional expression in unmedicated and medicated depressed participants. Table S4. Mean fixation time (in ms) per distractor face before target fixation as a function of target and crowd emotional expression in unmedicated and medicated depressed participants. Table S5. Mean fixation time (in ms) on target as a function of target and crowd emotional expression in unmedicated and medicated depressed participants. [file 12888_2021_3093_MOESM1_ESM.docx]

**Supplementary material**

Article title: **Efficient visual search for facial emotions in patients with major depression**

Authors: Charlott Maria Bodenschatz, Felix Czepluch, Anette Kersting, Thomas Suslow*

Charlott Maria Bodenschatz, Department of Psychosomatic Medicine and Psychotherapy, University of Leipzig, Semmelweisstraße 10, 04103 Leipzig, Germany, e-mail: charlott.bodenschatz@medizin.uni-leipzig.de

Felix Czepluch, Department of Social Psychology, University of Leipzig, Neumarkt 9-19, 04109 Leipzig, Germany, e-mail: felix.czepluch@uni-leipzig.de

Anette Kersting, Department of Psychosomatic Medicine and Psychotherapy, University of Leipzig, Semmelweisstraße 10, 04103 Leipzig, Germany, e-mail: anette.kersting@medizin.uni-leipzig.de

Thomas Suslow, Department of Psychosomatic Medicine and Psychotherapy, University of Leipzig, Semmelweisstraße 10, 04103 Leipzig, Germany, e-mail: suslow@medizin.uni-leipzig.de

^*^ Corresponding author

We conducted analyses on the impact of medication on reaction time and eye-tracking parameters in our patient group using 6 (condition) x 2 (group) mixed ANOVAs. The Greenhouse Geisser correction was applied to account for violations of sphericity where appropriate.

**Reaction time data**

ANOVA revealed a significant main effect of condition, *F*(5, 180) = 67.96, *p* < .001, $\eta_{p}^{2}$ = .65, but no main effect of group, *F*(1, 36) = .41, *p* = .53, and no significant interaction effect, *F*(5, 180) = 2.14, *p* = .06. Table S1 shows response latencies for both patient groups in the experimental conditions.

**Table S1. Reaction time (in ms) as a function of target and crowd emotional expression in unmedicated and medicated depressed participants.**

|  |  | uMDD (n = 14) | |  | mMDD (n = 24) | |
| --- | --- | --- | --- | --- | --- | --- |
|  |  | M | SD |  | M | SD |
| Angry target in happy distractors |  | 2038 | 373 |  | 1948 | 262 |
| Angry target in neutral distractors |  | 2653 | 381 |  | 2763 | 388 |
| Happy target in angry distractors |  | 2164 | 428 |  | 1992 | 379 |
| Happy target in neutral distractors |  | 2179 | 368 |  | 2088 | 429 |
| Neutral target in angry distractors |  | 2785 | 355 |  | 2822 | 476 |
| Neutral target in happy distractors |  | 2264 | 478 |  | 2046 | 453 |

Note: uMDD = unmedicated depressed participants; mMDD = medicated depressed participants; M = mean; SD = standard deviation.

**Eye-movement data**

**Latency to target**

ANOVA showed a main effect of condition, *F*(5, 180) = 4.97, *p* < .001, $\eta_{p}^{2}$ = .12, but no main effect of group *F*(1, 36) = 1.43, *p* = .24, and no interaction effect *F*(5, 180) = 1.01, *p* = .41. Latencies to target in the experimental conditions are presented for both patient groups in Table S2.

**Table S2. Latency to target (in ms) as a function of target and crowd emotional expression in unmedicated and medicated depressed participants.**

|  |  | uMDD (n = 14) | |  | mMDD (n = 24) | |
| --- | --- | --- | --- | --- | --- | --- |
|  |  | M | SD |  | M | SD |
| Angry target in happy distractors |  | 1218 | 171 |  | 1210 | 191 |
| Angry target in neutral distractors |  | 1386 | 282 |  | 1331 | 246 |
| Happy target in angry distractors |  | 1260 | 239 |  | 1236 | 203 |
| Happy target in neutral distractors |  | 1327 | 212 |  | 1305 | 247 |
| Neutral target in angry distractors |  | 1426 | 327 |  | 1296 | 193 |
| Neutral target in happy distractors |  | 1289 | 346 |  | 1131 | 202 |

Note: uMDD = unmedicated depressed participants; mMDD = medicated depressed participants; M = mean; SD = standard deviation.

**Number of distinct distractor faces fixated before fixating the target**

The results of the ANOVA indicated a main effect of condition, *F*(5, 180) = 4.04, *p* < .01, $\eta_{p}^{2}$ = .10, and a main effect of group *F*(1, 36) = 10.23, *p* < .01, $\eta_{p}^{2}$ = .22, but no interaction effect *F*(5, 180) = 1.76, *p* = .12. Number of distinct distractor faces fixated before target fixation in the experimental conditions are shown for the patient groups in Table S3. The present results suggest that medicated patients fixated fewer distractors before target fixation compared to unmedicated patients.

**Table S3. Number of distinct distractor faces fixated before target fixation as a function of target and crowd emotional expression in unmedicated and medicated depressed participants.**

|  |  | uMDD (n = 14) | |  | mMDD (n = 24) | |
| --- | --- | --- | --- | --- | --- | --- |
|  |  | M | SD |  | M | SD |
| Angry target in happy distractors |  | 3.15 | 0.34 |  | 3.00 | 0.61 |
| Angry target in neutral distractors |  | 3.27 | 0.69 |  | 3.14 | 0.58 |
| Happy target in angry distractors |  | 2.89 | 0.55 |  | 2.78 | 0.50 |
| Happy target in neutral distractors |  | 3.25 | 0.48 |  | 2.94 | 0.53 |
| Neutral target in angry distractors |  | 3.72 | 0.65 |  | 3.04 | 0.54 |
| Neutral target in happy distractors |  | 3.32 | 0.70 |  | 2.81 | 0.56 |

Note: uMDD = unmedicated depressed participants; mMDD = medicated depressed participants; M = mean; SD = standard deviation.

**Mean fixation time per distractor face before fixating the target**

The results of the ANOVA indicated a main effect of condition, *F*(5, 180) = 7.18, *p* < .001, $\eta_{p}^{2}$ = .17, but no main effect of group *F*(1, 36) = 0.56, *p* = .46, and no interaction effect *F*(5, 180) = 1.30, *p* = .27. Mean fixation times per distractor face prior to target fixation in the experimental conditions are presented for both patient groups in Table S4.

**Table S4. Mean fixation time (in ms) per distractor face before target fixation as a function of target and crowd emotional expression in unmedicated and medicated depressed participants.**

|  |  | uMDD (n = 14) | |  | mMDD (n = 24) | |
| --- | --- | --- | --- | --- | --- | --- |
|  |  | M | SD |  | M | SD |
| Angry target in happy distractors |  | 644 | 146 |  | 654 | 197 |
| Angry target in neutral distractors |  | 815 | 228 |  | 790 | 222 |
| Happy target in angry distractors |  | 693 | 198 |  | 717 | 200 |
| Happy target in neutral distractors |  | 760 | 149 |  | 733 | 224 |
| Neutral target in angry distractors |  | 866 | 251 |  | 758 | 179 |
| Neutral target in happy distractors |  | 729 | 253 |  | 621 | 199 |

Note: uMDD = unmedicated depressed participants; mMDD = medicated depressed participants; M = mean; SD = standard deviation.

**Mean fixation time on the target**

According to the ANOVA results there was a main effect of condition, *F*(3.41, 122.66) = 54.06, *p* < .001, $\eta_{p}^{2}$ = .60, but no main effect of group *F*(1, 36) = 0.15, *p* = .70, and no interaction effect *F*(3.41, 122.66) = 0.87, *p* = .47. Table S5 shows the mean fixation times on target as a function of experimental conditions and medication.

**Table S5. Mean fixation time (in ms) on target as a function of target and crowd emotional expression in unmedicated and medicated depressed participants.**

|  |  | uMDD (n = 14) | |  | mMDD (n = 24) | |
| --- | --- | --- | --- | --- | --- | --- |
|  |  | M | SD |  | M | SD |
| Angry target in happy distractors |  | 416 | 90 |  | 413 | 84 |
| Angry target in neutral distractors |  | 491 | 127 |  | 534 | 108 |
| Happy target in angry distractors |  | 413 | 75 |  | 389 | 71 |
| Happy target in neutral distractors |  | 367 | 69 |  | 378 | 87 |
| Neutral target in angry distractors |  | 620 | 83 |  | 654 | 165 |
| Neutral target in happy distractors |  | 450 | 112 |  | 446 | 100 |

Note: uMDD = unmedicated depressed participants; mMDD = medicated depressed participants; M = mean; SD = standard deviation.
